# Support or control? Qualitative interviews with Zambian women on male partner involvement in HIV care during and after pregnancy

**DOI:** 10.1371/journal.pone.0238097

**Published:** 2020-08-27

**Authors:** Karen M. Hampanda, Oliver Mweemba, Yusuf Ahmed, Abigail Hatcher, Janet M. Turan, Lynae Darbes, Lisa L. Abuogi

**Affiliations:** 1 Department of Obstetrics and Gynecology, School of Medicine, University of Colorado Anschutz Medical Campus, Aurora, Colorado, United States of America; 2 Center for Global Health, Colorado School of Public Health, University of Colorado Anschutz Medical Campus, Aurora, Colorado, United States of America; 3 Department of Health Promotion and Education, School of Public Health, University of Zambia, Lusaka, Zambia; 4 Department of Obstetrics and Gynaecology, Women and Newborn Hospital, University Teaching Hospitals, Lusaka, Zambia; 5 Department of Health Behavior, Gillings School of Global Public Health, University of North Carolina, Chapel Hill, North Carolina, United States of America; 6 Department of Health Care Organization and Policy, School of Public Health, University of Alabama at Birmingham, Birmingham, Alabama, United States of America; 7 Department of Health Behavior and Biological Sciences, Center for Sexuality and Health Disparities, School of Nursing, University of Michigan, Ann Arbor, Michigan, United States of America; 8 Department of Pediatrics, School of Medicine, University of Colorado Anschutz Medical Campus, Aurora, Colorado, United States of America; University of North Carolina at Greensboro, UNITED STATES

## Abstract

**Background:**

Efforts to promote male partner involvement in the prevention of mother-to-child transmission (PMTCT) may inadvertently exploit gender power differentials to achieve programme targets.

**Methods:**

We explored women’s relative power and perceptions of male partner involvement through interviews with postpartum Zambian women living with HIV (n = 32) using a critical discourse analysis.

**Results:**

Women living with HIV reported far-reaching gender power imbalances, including low participation in household decision-making, economic reliance on husbands, and oppressive gendered sexual norms, which hindered their autonomy and prevented optimal mental and physical health during and after their pregnancy. When the husband was HIV-negative, sero-discordance exacerbated women’s low power in these heterosexual couples. Male involvement in HIV care was both helpful and hurtful, and often walked a fine line between support for the woman and controlling behaviours over her. Inequities in the sexual divisions of power and labour and gender norms, combined with HIV stigma created challenging circumstances for women navigating the PMTCT cascade.

**Conclusions:**

Future programmes should consider the benefits and risks of male partner involvement within specific relationships and according to women’s needs, rather than advocating for universal male involvement in PMTCT. This work highlights the persistent need for gender transformative approaches alongside PMTCT efforts.

## Introduction

Commendable improvements have been made to provide prevention of mother-to-child transmission (PMTCT) services to all women living with HIV (WLWH) during and after pregnancy. As of 2017, 80% of pregnant WLWH globally had access to antiretroviral therapy (ART) through PMTCT programmes [1]. In the study setting of Zambia, access to lifelong ART for pregnant WLWH is estimated at over 90% through widespread and free government-run integrated HIV and antenatal care (ANC) [2].

In order to promote optimal maternal health and HIV-free infant survival, the PMTCT continuum requires successful behaviours during pregnancy and the postpartum period. Key behaviours across the PMTCT continuum include: maternal uptake and adherence to ART, retention in HIV care, safe infant feeding practices, providing infant HIV prophylaxis, and infant HIV testing [3, 4]. Similar to other sub-Saharan African settings [5–8], adherence and engagement across the entire PMTCT continuum in Zambia remain challenging [9–11], particularly during the postpartum period [8–10]. As such, reductions in new HIV infections in children have recently plateaued and many WLWH do not achieve optimal HIV outcomes like viral suppression during or after pregnancy [1].

Global leaders, such as the World Health Organisation (WHO), promote male partner involvement as a key strategy to improve PMTCT uptake and adherence [12, 13]. Recent literature, including several systematic reviews, report that male partner involvement–typically conceptualised as male attendance at ANC or couple HIV testing and counselling (CHTC)–is associated with improvements in women’s engagement in PMTCT [14–16]. In Zambia, the Ministry of Health has integrated CHTC into ANC [17] alongside various clinic and community outreach activities and incentives, such as jumping the queue if a woman presents with her male partner [18]. Within the promotion of male partner involvement in PMTCT, however, there is a lack of appreciation for the interplay of gender power dynamics. Through a critical discourse analysis of qualitative interviews with postpartum WLWH in Zambia, we explored how gender power imbalances affect women’s decision to involve male partners in HIV care and their perceptions of male partner involvement during the critical time periods of pregnancy and postpartum.

## Theoretical framework

Gender and power are interrelated factors known to influence health [19], including women’s risk of HIV [20]. According to the Theory of Gender and Power, there are three interrelated social structures operating across the social-ecology creating the gender order (i.e., global dominance of men over women): the sexual division of labour; the sexual division of power; and the structure of cathexis (i.e., gender norms) [21]. This patriarchal ordering of power and privilege is embedded in our historical, social, and political systems, and permeates into the family [22, 23]. As a result, women in many societies are often economically dependent on their male partners, expected to be subordinate, and have less household decision-making power [24, 25].

## Methods

### Overview

The goal of this qualitative study was to generate a better understanding of relationship processes with male partners that affect women’s PMTCT-related health behaviours through a critical examination of gender and power. This study was part of a larger concurrent mixed-methods parent study on the relationship between gender power dynamics within heterosexual couples and women’s PMTCT adherence. Details of the parent study have been published elsewhere [10]. Briefly, from March to August 2014, a cross-sectional survey was administered to 320 postpartum WLWH attending well-child paediatric healthcare visits at a large public health centre within a densely populated, low socio-economic neighbourhood of Lusaka. A convenience sub-sample of 32 participants in the parent study was invited to also participate in a semi-structured qualitative interview. The goal of the interviews was to expand on and explain the quantitative survey findings regarding the relationship between gender power dynamics and PMTCT-related health behaviours.

At the time of the study (2014), Zambia was transitioning to the national policy of “Option B+” (lifelong ART for all pregnant WLWH). Thus, participants in the study included both women who were initiated on lifelong ART, as well as women who were prescribed short-course antiretroviral regimens who did not meet the criteria for lifelong ART under the former “Option A” health policy. Both policies included infant HIV prophylaxis but for different lengths of time. The infant feeding recommendation for all WLWH at the time of data collection was, and still is, to exclusively breastfeed to six months and to continue breastfeeding to 12 months or longer with complementary food. Per country guidelines, all HIV-exposed infants should be tested for HIV at six weeks, six months, 12 months, and 18 months, and immediately initiated onto treatment if HIV-positive [3].

### Participant sampling and recruitment

Participants were recruited during routine paediatric healthcare visits (e.g., child immunisations, height and weight measurements). Women were eligible for participation if they were married or cohabiting with a male partner, HIV-positive, over 18 years of age (legal age to provide consent for research in Zambia), and had a biological child between 3 to 9 months of age. Infant age criteria were meant to capture the essential PMTCT protocols, match the paediatric immunisation schedule, and limit recall bias. Because a major focus of the parent study was on intimate partner violence (IPV), as a safety measure, we excluded any women who were at the clinic with their male partners; only one woman was excluded for this reason. Nurses at the clinic determined eligibility for the parent study using the child’s “Under-Five Card” (i.e., a mother’s copy of her child’s health record that she is required to bring to all healthcare visits) or other available medical records. Eligible women were consented by research staff and received a small travel reimbursement.

All survey participants were invited to stay and participate in a semi-structured interview immediately after the survey on the same day in the same location. Interviews were conducted by experienced, trained local Zambian research assistants in the most commonly spoken languages (English, Nyanja, Bemba, Tonga) using a semi-structured interview guide (see S1 File). The interview guide included broad, open-ended questions regarding PMTCT experiences and gender power dynamics. All research assistants had qualitative public health experience and participated in a three-day training. Data analysis and recruitment occurred concurrently and continued until the research team agreed we had achieved theoretical saturation of themes informing *how* gender power dynamics affect women’s PMTCT-related health behaviours. Throughout data collection, memos were kept in order to create a rich description of the data and to identify any needed changes to the interview guide, as well as establish theoretical saturation. Interviews were audio-recorded, translated and transcribed verbatim into Microsoft Word, and imported into Atlas.ti for analysis.

### Critical discourse analysis

The codebook (see S2 File) was developed and applied to the transcripts by the first author (KH) using a combination of a priori codes from the interview guide and emergent codes. The author began with initial, line-by-line coding of transcripts to identify meanings and assumptions within the data, as well as comparisons between the codes and participants [26]. In the final stages of analysis, focused coding by the first and second authors (KH and OM) explored the underlying meanings of the participant narratives and how they add to, form, transform, or reflect gendered social structures and processes in relation to women’s HIV care during and after pregnancy [27]. We applied Fairclough’s method of critical discourse analysis, which emphasises how participant narratives are linked to societal and cultural processes and structures [28]. Our critical discourse analysis interrogated the transcripts by paying attention to issues of explicit and implicit gender power dynamics [29] and how participants navigated these in the context of PMTCT care.

### Ethical considerations

The study was approved by relevant ethics committees at the Colorado Multiple Institutional Review Board (protocol # 13–1979), and the Excellence in Research Ethics and Science Converge Institutional Review Board in Zambia (protocol # 2014-Jan-010). All participants provided written informed consent. Respondents' identities were protected by (1) deidentifying all transcripts and assigning a study number prior to analysis; and (2) using pseudonyms/false names when reporting results.

## Results

### Socio-demographics and gender power dynamics

Table 1 presents the sample characteristics of the 32 interview participants. On average, the WLWH in this study were 27 years of age with three children. The majority (68%) had completed a primary education, but only 19% had achieved secondary education. The sample had relatively low socioeconomic status, indicated by a mean of only 7 household items, such as electricity or a television [30]. The majority of participants (79%) reported employment outside of the home. WLWH reported relatively high levels of ART adherence (>90%) based on a visual analogue scale with an adherence cut-off of taking 80% of prescribed doses [31]. Almost one-quarter of the couples were HIV sero-discordant (male partner HIV-negative) with the majority of women (80%) reporting HIV status disclosure to the male partner.

**Table 1:** Sample characteristics (n = 32).

Variable	Descriptive statistic
Woman’s age (years): mean	26.9
Infant age (months): mean	4.8
Parity: mean	2.8
Completed primary education (yes): %	68%
Completed secondary education(yes): %	19%
Number of household assets:^1^ mean	6.7
Employment outside of the home in the past 12 months (yes): %	79%
Maternal ART adherence (yes):^2^ %	91%
Discordant couple (yes): %	23%
Disclosed HIV status (yes): %	80%
Gender power dynamics:	59%
Intimate partner violence:	
Any IPV (yes): %	
Any emotional IPV (yes): %	37%
Any physical IPV (yes): %	40%
Any sexual IPV (yes): %	30%
Male controlling behaviours:	3.2
Always jealous (yes): %	73%
Frequently accuses you of being unfaithful (yes): %	63%
Does not permit you to meet female friends (yes): %	57%
Tries to limit your contact with family (yes): %	37%
Insists on knowing where you are at all times (yes): %	50%
Does not trust you with money (yes): %	27%
Women’s participation in decisions about her healthcare (yes):^3^ %	53%
Women’s relative earnings:	
Less than husband	47%
Equal to husband	20%
Greater than husband	10%
Not sure	23%
**Total**	100%

^1^ From a list of 21 items (based on Zambian Demographic and Health Survey).

^2^ Postpartum ART adherence measured via self-report on the survey using a visual analogue scale.

^3^ Women reporting they alone or together with the husband made the decision about her use of healthcare. Comparison group: the decision is made by the husband alone.

Gender power dynamics were readily observed with almost 60% of participants reporting some form of IPV (emotional, physical, or sexual violence) in the relationship. In addition, male controlling behaviours were commonly reported with the majority of participants reporting the male partner is always jealous (73%); frequently accuses her of being unfaithful (63%); and limits contact with her friends (57%). Close to half of the women (47%) reported no participation in decisions regarding their own healthcare. Economic disparities were prevalent with 47% of women reporting less earnings than the husband.

In the following sections, we present key qualitative themes related to women’s journey through HIV care and treatment during and after pregnancy and their views on male partner involvement.

### The decision to disclose

Among our participants, HIV status disclosure to husbands followed different pathways closely tied to gender power dynamics. These dynamics were exacerbated by persistent HIV-related stigma and inequitable sexual norms, where WLWH feared connotations of infidelity and promiscuity. Participants explained how they often carefully considered disclosure risks and benefits depending on characteristics within their relationship. The fear of marriage dissolution was ubiquitous among women who made the decision to hide their HIV status. As one 23-year-old-participant, Judith, explained: “it was difficult for me to tell my husband my HIV status because I thought he is going to divorce me.” Participants who made the decision to hide their status from their husband repeatedly justified their decision based on their economic dependence on the partner–a clear consequence of the sexual division of labour. The following quote from Eve highlights how her decision to hide her status was based on fears of abandonment, combined with the man’s projected HIV-related stigma (the dominant voice in her narrative). Eve thus concluded the consequences of disclosing her positive HIV status were too great because it would lead to hardship in her ability to care for her children:

“…*there is no way I could have disclosed to him my HIV status…I was just scared because he always says that if one of us were HIV positive it would be the end of our marriage and I was scared that how could I look after our children on my own*?”

–Eve, 29 year-old-woman

The presence of IPV–a compelling manifestation of the sexual division of power–was additionally discussed as a factor that prevented WLWH from disclosing their HIV status. The following quote from Ruth highlights how her past experiences of IPV and fear of abandonment led her to avoid status disclosure to her abusive partner in order to ensure her safety:

“*I think it is better to continue hiding my HIV status from my husband because if I told him he would chase me from our home… I am not prepared to stay alone*. *This man has a hot temper*, *when he is angry*, *he always beats me up* …”

-Ruth, 25 year-old-woman

Similar to Eve, Ruth feared that telling her abusive partner about her positive HIV status would cause him to kick her out of the marital home, meaning she would either need to return to her maternal home or somehow find money to live on her own. Ruth described that she did not feel she would be able to care for her children alone without the resources that were provided to her (e.g., their home and money) by staying married. These narratives reflect how HIV stigma, oppressive gender norms, and women’s dependence on male partners led to a perceived social reality where WLWH fear status disclosure. In the following section, we highlight the consequences of non-disclosure.

### The consequences of non-disclosure

Among the participants who hid their HIV-positive status from their husband, PMTCT was more arduous by, for example, having to hide medication, lie about its purpose, or sneak to HIV care. However, this decision was often justified based on the perception that the outcome of the partner knowing the woman’s positive HIV status would be more detrimental to their and the children’s well-being. For instance, Dinah admitted that her ART adherence was negatively affected by non-disclosure but that she could not disclose her status to her husband because she, like many others, feared abandonment due to HIV stigma:

“*It was difficult for me to take the medication because I didn’t want my husband to see me taking it*. *I am scared it might end my marriage if he discovered I am HIV-positive*. *I just hear people that say that having the virus it is better to just die* …”

–Dinah, 34 year-old-woman

A compounding factor in Dinah’s situation was that her husband informed her that he tested for HIV at work and was found HIV-negative. Indeed, sero-discordance emerged as an important theme exacerbating women’s low perceived power.

Another example of the far-reaching consequences of non-disclosure, closely linked to gender power dynamics, is described by Eve, who struggled with taking ART and attending clinic appointments due to her husband’s controlling behaviours along with her fear of status disclosure:

“*I was not bringing my child to the clinic because I was scared of my husband*. *From the time I was pregnant*, *I have been taking ART*, *but I have not told my husband that I am taking it*. *It has been tough for me*. *Now there are times that I even miss my appointments because I did not find chance to sneak to the clinic*. *Every time I want to take my child to the clinic*, *he always gives me problems… He lingers in the house and will not leave the house until he is given something to eat*. *That is when I can find time to leave*. *Even these scars that you see on my face*, *he really beats me up over such issues*. *It has not been easy for me*. *At some point I even stopped taking my drugs for sometime*.”

–Eve, 29 year-old-woman

Eve’s narrative explains how gender power inequities in the family (e.g., male controlling behaviours, expectations of women’s domestic labour, and IPV), coupled with a lack of status disclosure, can significantly limit women’s ability to engage in optimal PMTCT and cause significant distress.

Another participant, Sylvia, similarly discussed the consequences of non-disclosure where she found PMTCT challenging because she had to hide her ART from the husband. Like many others, Sylvia’s narrative closely overlapped with discussions of HIV stigma, as well as poor mental health:

“*I used to have problems when I started ART* … *I worried where am I going to put this medication where my husband will not see it*… *I sometimes stopped taking because I used to just feel so sad when I looked at my baby and me and think it would be better for both of us to just die than to live with HIV*.”

- Sylvia, 25-year old woman

The narratives discussed above help explain *why* non-disclosure can lead to poor PMTCT outcomes. Yet, we also discovered that disclosure did not universally result in positive outcomes among our participants either, as described in the following sections.

### The consequences of disclosure

Although not universal, several of our participants, especially those in sero-discordant relationships, reported a negative reaction after HIV status disclosure to the male partner, including IPV, which impaired their mental health and the ability to engage in optimal PMTCT. First, several women discussed emotional abuse in the form of the husband belittling them for having HIV and accusing them of being promiscuous (i.e., violating women’s sexual norms). Belinda, for instance, explained how she experienced constant questioning from her HIV-negative husband about how she acquired HIV. Their sero-discordance led to the husband being resistant about PMTCT, which Belinda felt she did not have the power to contest:

“*I have problems at home with my husband because he insists to ask questions like ‘what happened to you*, *how can you be [HIV] positive and I am negative’ and ‘where did you get this infection from that has made you start giving this medication to the baby*?*’ I have tried to explain but it seems he does not understand and does not like me giving HIV prophylaxis to the baby… it is not easy*.”

−Belinda, 34 year-old-woman

Not only did Belinda experience difficulties with the partner accepting her HIV status, but she also found it difficult to act independently and give the child HIV prophylaxis in her husband’s presence, even though she clearly was aware that prophylaxis is an important component of PMTCT.

Similar to Belinda’s experience, after status disclosure, Judith’s husband was judgmental around the couple’s sero-discordancy, accusing her of infidelity, which in turn, affected Judith’s quality of life and made ART adherence difficult:

“*I was not taking the medication because I was scared of my husband*. *I just used to hide the medication because he does not like me taking it*. *He wants me to stop the medication and instead pray and go see the pastor*. *My husband is very stubborn and does not want to listen*. *That man is really making my life very hard*. *It is difficult for me to take the medication because he is always tormenting me on the same issues every day [sero-discordance] …He tells me that it seems I started a long time ago sleeping around*.”

–Judith, 23 year-old-woman

Women described how their economic reliance on husbands created distressing situations in sero-discordant relationships when the partner was unsupportive of the woman’s HIV status. For example, Marie, who had disclosed her status to her HIV-negative husband, described financial abuse and hardship:

“*When he was paid*, *he would miss from home for sometime and when the money was finished that is when he would come back home*. *When you ask him why*, *he would say you*, *‘you are sick*, *me I am not*.’”

–Marie, 21 year-old-woman

Some participants reported they were unaware of the husband’s HIV status because he refused to test. This highlights another key aspect of gender power dynamics: the inability to control the husband’s HIV-related behaviours. In the case of Patricia, not only did her husband refuse to test, but she believed he was the source of her HIV infection:

“*He just came back home one day and told me ‘I am a polygamist; I have another wife somewhere so there will be the two of you*.*’ I could have died… I became too angry with him because to the best of my knowledge*, *it is him that infected me with HIV and then he started teasing me with his girlfriends… I don’t know anything about his status because he is one man that refuses to get tested*.”

–Patricia, 24 year-old-woman

When Patricia disclosed her positive HIV status to her partner, she recalled how he began spending long periods away from home with other sexual partners, and that eventually, he moved out of their home. His lack of support, as well as Patricia’s lack of income, caused her significant mental distress:

“*He did not even want to listen to anything concerned with HIV*. *No wonder that from that time I was diagnosed I had so many thoughts because he did not show any support for me in that issue [HIV] that I had brought home (crying)*. *He started even sleeping out [with other women] at that time*. *He would go out for a long time without coming home* …*now I am living at home alone with my child and I am not doing anything for a living*.”

–Patricia, 24 year-old-woman

In the following section, we describe how other participants received a more positive reaction from male partners to their HIV status disclosure, which led to improved coping, better mental health, and improved PMTCT adherence. Yet, even in the narratives depicting male “support,” a critical discourse analysis reveals persistent undertones of gender power dynamics.

### Support for or control over women?

Some women discussed a positive reaction after disclosure of their HIV-positive status to the husband, including the provision of various forms of emotional and instrumental support. These instances were notably mentioned by women in both sero-discordant and concordant relationships, and in cases where the husband’s status was unknown. Women credited such positive interactions in reducing internalised HIV stigma, improving their ability to cope with their HIV diagnosis, and facilitating PMTCT-related engagement. For example, Monica, whose partner’s HIV status was unknown, described her initial challenges with adherence and depression, but how her reluctance to start ART was soothed by her husband’s encouragement:

“*When I was diagnosed with HIV it pained me a lot*, *I didn’t even eat*. *At first I had the attitude I would never take the medicine*. *I would just die like that*. *But my husband has been encouraging me…*. *he is the one that convinced me to start taking the medication*.”

- Monica, 27 year-old-woman

Similarly, Chelsey, whose husband was also living with HIV, credited the support she received from him for her ability to be adherent to ART and for her children’s health:

“*When we went to the clinic*, *they told us we were [HIV] positive*. *For me it was tough to accept that I have HIV*, *but for my husband*, *he was the one that encouraged me and told me it is going to be okay*. *He is a good husband for doing that*. *If you have someone to encourage you*, *things will go well*. *I don’t forget to take medication now and all of my children have been born without any issues [HIV-negative]*.”

–Chelsey, 29-year-old woman

Without discrediting these provisions of support, our analysis also highlights the concomitant presence of gender power dynamics. For instance, Chelsey’s statement above about her partner being a “*good husband”* indicates that she expected a different, more negative reaction. Among the women in sero-discordant relationships who discussed supportive partners, there was a heavy focus on “being grateful” that the husband wanted to stay with them after learning they have HIV. Moreover, women often depicted the decision to stay together after HIV status disclosure as being primarily, if not entirely, up to the husband.

In the case of Alice, she stated that her husband was supportive regarding her HIV diagnosis, but seemed to have had little participation in the decision for the couple to stay together after learning of their sero-discordance:

“*He was just so supportive*, *he encouraged me to take the drugs the way I was told at the clinic*. *He encouraged me that ‘you are not the first one to be found with HIV… I have already stayed with you for a long time so let’s just continue to stay together despite one of us having HIV*.’”

−Alice, 20 year-old-woman

The quote below from Phoebe presents a unique narrative of how some women expressed perceptions of autonomy over their health care decisions, while simultaneously surrendering power in relationship decisions to the husband. Phoebe stated that she would not want to stay with her husband if he discouraged her from taking ART due to the risk of mother-to-child transmission, but that she would need to ask *him* to end the marriage:

Interviewer: *“What if your husband told you to stop taking the drugs*?*”*

Participant: *“For me*, *that would be the end of our marriage*. *I would really ask him to end the marriage because I would not be happy for my baby to be HIV-positive and have to take ARVs for life like me* …*”*

- Phoebe, a 20-year-old

In several interviews, it was unclear whether the husband’s behaviour should be classified as supportive or controlling, as in the case of Monica:

“*When it came to taking the medicine*, *I didn’t want to do it*, *but my husband*, *he is the one that convinced me to start*. *He sat down next to me and forced me to drink the medication while he watched me until I found the courage to drink on my own*. *Even up until now he still asks me if I have been taking the medication*. *He notices all the time when I am taking the medicine*. *He says I might be throwing the medicine in the toilet*, *so he sits with me and watches me take the medicine*. *Sometimes I try to lie and say I have taken the medication (laughs)*, *but he is like ‘why haven’t I seen you take the medication*, *take it now*.’”

- Monica, 27 year-old-woman

The above quote highlights how women’s PMTCT-related health behaviours are affected by the sexual division of power where a woman’s decision to take ART may not be her own. Monica clearly believed that her husband had the authority to make decisions about *her* HIV-related care and that she is accountable to him. Her narrative and tone also indicated that she perceived this male authority to be acceptable and beneficial.

### Resilience and resistance

A final theme that arose in our interviews was women’s remarkable resilience to adverse situations and, in some instances, resistance to gender-based power structures. For example, many of the women we interviewed who were fearful to disclose their HIV status to the husband, reported using creative ways to act independently and engage in PMTCT. Eve described how despite having a physically abusive, highly controlling husband who was unaware of her HIV status, she still found ways to engage in HIV care:

*For me to find time to come to the clinic*, *I have to make sure that he has gone somewhere very far and that is how I sneak and come to the clinic to collect my drugs*.”

–Eve, 29 year-old-woman

In another interview, Ruth described how her HIV diagnosis and disclosure to an ex-husband had ended her first marriage, which led her to fear disclosure to the current husband. Despite a lack of disclosure, Ruth explained how she was able to maintain high adherence by taking her ART in secret and deceiving her husband:

“*I am scared of being chased from my home again so I have continued hiding my medication from him*. *I always take the medication when he has gone for work… With the baby*, *it is not difficult*, *because what he thinks is that I am giving a pain killer to the baby*. *There has never been a day that I have missed giving medication to the baby*. *I know that this medication is protecting my baby from getting infected*.”

-Ruth, 25 year-old-woman

Other narratives detailed how women were able to use past experiences of trauma to motivate resistance against gender power dynamics and engage in health enhancing behaviours. For instance, several women had experienced the death of an older child. These participants universally expressed how they were highly motivated to be adherent to PMTCT with their current infant because of their mothering role and the intense remorse from the prior child’s death. For instance, Judith explained that she was able to be adherent despite ongoing emotional abuse and objections from her husband because she desperately wanted to prevent another child death:

“*I blamed myself for my first child passing away*, *so even though my husband used to call me names*, *he called me a prostitute because I was HIV-positive*, *I still used to hide from him and come here to get the medication to protect my unborn child from HIV*. *I saw what happened with my other child–he was almost one year*, *but then he passed away… so now even if my husband tries to prevent me from going to HIV care*, *I still go and get the medication and give to my child*.”

- Judith, 23 year-old-woman

The above narratives illustrate that despite widespread gender inequities, WLWH can display remarkable resilience against the gender order in an effort to protect their children from HIV.

## Discussion

This study revealed the multiple layers of gender inequities that women must navigate in their journey through HIV care during and after pregnancy. Gender inequities and sexual norms coupled with HIV stigma, hindered status disclosure for many women, which made ART adherence and HIV care more challenging. Even among women who disclosed their HIV-positive status to the husband, there were major barriers when male partners reacted negatively. In situations where the male partner was accepting of the woman’s positive HIV status, women’s depictions of male support still had strong undertones of male control with shared decision-making absent in their narratives. Throughout the interviews, there was the persistent theme of women’s lack of autonomy in decisions, including whether the relationship should continue or their use of PMTCT.

Prior literature indicates that women’s disclosure of an HIV-positive diagnosis to male partners is linked to improved HIV care and treatment around the time of pregnancy [10, 32, 33]. This study revealed, however, why status disclosure needs to be examined within a framework of gender and power. Our findings affirm and help explain the barriers to HIV status disclosure to male partners that have been reported by women across various sub-Saharan African settings, such as fears of divorce and violence [34–39].

Given the high social importance of marriage for women in settings like Zambia (i.e., structure of cathexis), and often, economic dependence on the partner (i.e., the sexual division of labour) [40], fears of divorce and abandonment were an extremely powerful motivating factor to hide their HIV status, even if it meant difficulties with PMTCT. Indeed, in patrilineal and patrilocal societies, such as Zambia, women have very little property ownership and in the event of divorce, traditional law dictates the man owns and keeps all property/assets [41]. The presence of IPV (i.e., the sexual division of power) [42] was an additional factor that prevented women from disclosing their HIV-positive status. Underlying the fear of violence or abandonment was, for many women, a fear of being accused of violating sexual norms dictating female purity and fidelity (i.e., structure of cathexis) [25]. Lastly, similar also to other studies in the region [43, 44], our analysis found that sero-discordance when the woman is HIV-positive was an important theme exacerbating women’s low power in the relationship.

Unfortunately, some women’s fears about status disclosure were real occurrences for the women who chose to disclose their HIV-positive status. In relationships characterised by greater gender power inequities, such as when IPV was present, women often perceived little HIV-related partner support after disclosure. Women discussed how this contributed to difficulty coping with their HIV infection, caused significant mental health distress, and made PMTCT challenging. This finding is supported by other qualitative work from the region indicating a connection between gender power dynamics like IPV, women’s poor mental health (e.g., depression and anxiety), and sub-optimal PMTCT adherence [39]. In our narratives, women often viewed male partner involvement post-disclosure as unhelpful, or even hurtful, supporting existing literature questioning if status disclosure to male partners should *always* be promoted [38, 45].

Even in relationships that were perceived to be more positive, there was a fine line between support and control by male partners. Similar to prior research [46–52], for some of the WLWH we interviewed, perceived social support from the husband led to improved self-esteem and seemed to mediate the effects of stress associated with having a stigmatised, chronic disease like HIV, leading to improved PMTCT. Our critical discourse analysis highlighted, however, a number of incidences of high levels of male control over women’s health care. Indeed, many of the WLWH in this study struggled to act independently around medical decisions, including ART, which has also been documented in other African settings [53–55]. This is not necessarily surprising given the dominant role many husbands play in household decision-making (i.e., sexual division of power) in setting like Zambia [30]. Indeed, inequitable gender norms (i.e., the structure of cathexis) in such settings have traditionally promoted the idea of wives being subordinate to their husbands; many Zambian women report being counselled upon reaching puberty to be submissive and obey their future husbands [56]. It was not uncommon for WLWH in this study to report that husbands had the authority to make decisions about *women’s* HIV-related care and that this male authority is acceptable and beneficial.

In this study, we also documented resistance to the gender order, which was reported by WLWH who were acting autonomously in an effort to protect their children from HIV. This finding offers support for other qualitative research on the important role of women’s motherhood identity for PMTCT [39]. Indeed, our interviews found laudable instances of resilience among WLWH who resisted gender power dynamics that could hinder their own and their children’s health. More research is needed to better understand how to enhance such resilience among WLWH to enhance PMTCT efforts.

Our findings point to the complexities of gender power dynamics in the context of male involvement in PMTCT. Indeed, other scholars have questioned whether involving men in health care around the time of pregnancy may aggravate male control and dominance in an arena that has typically been under female control [57]. Efforts to involve male partners in PMTCT in settings like Zambia need to be cognisant about the potential of inadvertently exploiting gender power differentials to achieve HIV prevention and treatment goals. Sustainable social change and health equity will only be realised if male involvement in PMTCT is promoted alongside gender transformative efforts, including women’s empowerment and participation in medical decision-making [44].

The findings of this study should be interpreted within its limitations and strengths. First, the sample of postpartum WLWH who participated in the qualitative interviews was from a low socio-economic urban area in the capital city of Zambia and recruited from a clinic-based setting. Thus, our sample may not be representative of other populations of WLWH. We do not have the perspective of male partners and recommend future research to try and capture their experiences and perceptions. The exclusion of women who were at paediatric healthcare with their male partners may have biased the sample towards participants with less supportive partners. However, we found a wide spectrum of partner support and only one participant was excluded for this reason. Social desirability bias and interviewer bias may be present despite thorough training of the research team. Our participants, although sampled conveniently, included a diverse sample of women with heterogeneous experiences around gender power dynamics, status disclosure, and PMTCT adherence. This enabled us to capture a depth of information and expand our understanding of how gender power dynamics affect PMTCT in a high HIV-prevalence urban setting of Zambia. This has important implications for ongoing efforts to involve men in PMTCT and reduce gender inequities.

## Conclusions

Our findings highlight why male partner involvement in PMTCT, which by definition, requires women to disclose their HIV-positive status, is not always feasible nor helpful. We uncovered key aspects related to gendered structures in the lives of woman with HIV–often full of contradictions–they endured while navigating HIV care and treatment during and after a pregnancy. We learnt that for many women, there was a fine line between male partner support and male controlling behaviours, highlighting the sexual division of power where women perceived male partners as having authority over their HIV care. While involving men in PMTCT efforts certainly has health-promoting potential, effort should be made to facilitate male partner involvement alongside gender equity and shared decision-making.

## Supporting information

S1 File (DOCX)

S2 File (DOCX)
