# Supplementary material for: Support or control? Qualitative interviews with Zambian women on male partner involvement in HIV care during and after pregnancy
Source: PLoS One. 2020 Aug 27;15(8):e0238097. doi: 10.1371/journal.pone.0238097 (PMC7451516; doi:10.1371/journal.pone.0238097)
Supplement: S1 File — (DOCX) [file pone.0238097.s001.docx]

**Semi-Structured Interview Guide**

Please put a star next to any questions/topics that the interviewee provided interesting details or things that are especially noteworthy.

***Mother PMTCT adherence (refer back to the questionnaire):***

***very good good poor very poor***

1. Why would you say that it was difficult to take medication every day the way you are supposed to during pregnancy/postnatal?

____________________________________________________________________________________________________________________________________________________________________________________________________________________________________________________________________________________________________________________________________________________________________________________________________________________________________

1. Why would you say that it was difficult to give the ARV medication every day to the child exactly the way you were told?

____________________________________________________________________________________________________________________________________________________________________________________________________________________________________________________________________________________________________________________________________________________________________________________________________________________________________

1. What has helped you be able to take your medication/give the medication to the child on time every day? (what skills does the mother use to remember to take/give the medicine – alarm? takes with husband? etc.)

____________________________________________________________________________________________________________________________________________________________________________________________________________________________________________________________________________________________________________________________________________________________________________________________________________________________________

***Has the mother introduced other food before 6 months (refer back to questionnaire)?***

***yes no***

1. Why did you decide to introduce (or not) to introduce foods before 6 months to you baby? Did anyone influence this decision?

____________________________________________________________________________________________________________________________________________________________________________________________________________________________________________________________________________________________________________________________________________________________________________________________________________________________________

1. How does your husband/partner feel about PMTCT, such as taking medication or giving medication to the infant?

____________________________________________________________________________________________________________________________________________________________________________________________________________________________________________________________________________________________________________________________________________________________________________________________________________________________________

***Has mother disclosed to partner (refer back to questionnaire) yes no***

1. Why did you decide (or not) to disclosure your HIV status to your husband/partner?

____________________________________________________________________________________________________________________________________________________________________________________________________________________________________________________________________________________________________________________________________________________________________________________________________________________________________

1. How did your husband/partner react when you told him? (OR how do you think he would react if you told him)

____________________________________________________________________________________________________________________________________________________________________________________________________________________________________________________________________________________________________________________________________________________________________________________________________________________________________

1. Would it have been easier to take medication or give medication to the infant if your husband/partner were not present?

____________________________________________________________________________________________________________________________________________________________________________________________________________________________________________________________________________________________________________________________________________________________________________________________________________________________________

1. What would happen if you disobeyed your husband/partner?

____________________________________________________________________________________________________________________________________________________________________________________________________________________________________________________________________________________________________________________________________________________________________________________________________________________________________

1. Could you leave your husband if you wanted to? Why or why not?

____________________________________________________________________________________________________________________________________________________________________________________________________________________________________________________________________________________________________________________________________________________________________________________________________________________________________

**Any other questions asked/topics covered:**

1. __________________________________________________________________________________________________________________________________________________________________
2. __________________________________________________________________________________________________________________________________________________________________
3. __________________________________________________________________________________________________________________________________________________________________
4. __________________________________________________________________________________________________________________________________________________________________

**Additional Comments about the interview:**

________________________________________________________________________________________________________________________________________________________________________________________________________________________________________________________________________________________________________________________________________________

____________________________________________________________________________________

**Topics that came up in the interview that would be worth following-up on during the next interviews:**

1. _________________________________________________________________________________
2. _________________________________________________________________________________
3. _________________________________________________________________________________
4. _________________________________________________________________________________
