# Supplementary material for: Support or control? Qualitative interviews with Zambian women on male partner involvement in HIV care during and after pregnancy
Source: PLoS One. 2020 Aug 27;15(8):e0238097. doi: 10.1371/journal.pone.0238097 (PMC7451516; doi:10.1371/journal.pone.0238097)
Supplement: S2 File — (DOCX) [file pone.0238097.s002.docx]

| **Code** | **Definition** |
| --- | --- |
| Adherence | Referring to PMTCT-related health behaviours (clinic attendance, ART, infant prophylaxis, infant feeding, infant testing) and adherence to recommended protocols. Also includes factors influencing ability to be adherent to PMTCT. |
| Child death | When death of a biological child is referenced |
| Disclosure | Whether the woman has told the partner that she is living with HIV, her decision-making process, her perceptions about disclosure, and her experiences with disclosure to the male partner |
| HIV status | Women’s perceptions of having HIV, coping, and stigma around living with HIV |
| Sexual division of power | Gender power dynamics in the relationship related to women's participation in decision-making, the use of violence, the ability to act independently; and who is accountable to whom |
| Sexual division of labour | Gender power dynamics in the relationship related to economics, household labour, and dependence on a partner for financial support |
| Structure of cathexis | Gender power dynamics related to expected gender norms (e.g., female subordination), including sexual norms (e.g., female purity and monogamy) |
| Control | Male partner control, including controlling or attempting to control the woman’s health decision making; limiting access to transportation, money, friends, and family; excessive monitoring of the woman’s behaviour and/or whereabouts |
| Support | Provisions of support by the male partner, including emotional (e.g., reassurance, acceptance, love) and/or instrumental (e.g., reminders to go for HIV care, transport money, collecting medication, etc) |
| Emotional abuse | The use of verbal and/or non-verbal communication with the intent to harm the woman mentally or emotionally, including e*xpressive aggression* (e.g., name-calling, humiliating, degrading, acting angry in a way that seems dangerous); threats of physical violence; exploitation of vulnerability |
| Physical abuse | Incidences of the male partner hurting or trying to hurt the woman by hitting, kicking, or using another type of physical force, including scratching, pushing, shoving, throwing, grabbing, biting, choking, shaking, hair-pulling, slapping, punching, hitting, burning, use of a weapon (gun, knife, or other object), or using another type of physical force |
| Partner status | Whether the husband is HIV-positive, HIV-negative, or has an unknown status and any experiences or perceptions related to his status |
| Relationship factor | Other dynamic in the relationship not captured by other codes, such as being married or not, length of relationship, level of satisfaction, communication in the couple, and conflict resolution |
| Mental health | Women’s reference to their mental and emotional well-being (e.g., feeling sad) |
| Resistance | Behaviours that reflect women’s resistance to gender power dynamics (e.g., taking ART when the husband did not want her to) |
| Mothering role | Women’s discussions of the importance of being a mother and prioritizing their children |
| Resilience | Reference to the ability to cope with hardship and achieve desired outcomes despite barriers |
